# Reducing intrapartum fetal deaths through low-dose high frequency clinical mentorship in a rural hospital in Western Kenya: a quasi-experimental study

**DOI:** 10.1186/s12884-019-2673-0

**Published:** 2019-12-23

**Authors:** Duncan N. Shikuku, Rita Mukosa, Taphroze Peru, Alice Yaite, Janerose Ambuchi, Kenneth Sisimwo

**Affiliations:** 1 Save the Children, Busia, Kenya; 2 Department of Health, Busia, Kenya

**Keywords:** Clinical mentorship, Low-dose high-frequency, Intrapartum fetal death, Midwife-led

## Abstract

**Background:**

Intrapartum fetal mortality can be prevented by quality emergency obstetrics and newborn care (EmONC) during pregnancy and childbirth. This study evaluated the effectiveness of a low-dose high-frequency onsite clinical mentorship in EmONC on the overall reduction in intrapartum fetal deaths in a busy hospital providing midwife-led maternity services in rural Kenya.

**Methods:**

A quasi-experimental (nonequivalent control group pretest – posttest) design in a midwife-led maternity care hospitals. Clinical mentorship and structured supportive supervision on EmONC signal functions was conducted during intervention. Maternity data at two similar time points: Oct 2015 to July 2016 (pre) and August 2016 to May 2017 (post) reviewed. Indicators of interest at Kirkpatrick’s levels 3 and 4 focusing on change in practice and health outcomes between the two time periods were evaluated and compared through a two-sample test of proportions. Proportions and *p*-values were reported to test the strength of the evidence after the intervention.

**Results:**

Spontaneous vaginal delivery was the commonest route of delivery between the two periods in both hospitals. At the intervention hospital, assisted vaginal deliveries (vacuum extractions) increased 13 times (0.2 to 2.5%, *P* < 0.0001), proportion of babies born with low APGAR scores requiring newborn resuscitation doubled (1.7 to 3.7%, *P* = 0.0021), proportion of fresh stillbirths decreased 5 times (0.5 to 0.1%, *P* = 0.0491) and referred cases for comprehensive emergency obstetric care doubled (3.0 to 6.5%, *P* < 0.0001) with no changes observed in the control hospital. The proportion of live births reduced (98 to 97%, *P* = 0.0547) at the control hospital. Proportion of macerated stillbirths tripled at the control hospital (0.4 to 1.4%, *P* = 0.0039) with no change at the intervention hospital.

**Conclusion:**

Targeted mentorship improves the competencies of nurse/midwives to identify, manage and/or refer pregnancy and childbirth cases and/or complications contributing to a reduction in intrapartum fetal deaths. Scale up of this training approach will improve maternal and newborn health outcomes.

## Introduction

Approximately three – quarters of all neonatal deaths occur during the first week of life, with a million babies dying on the day they are born [1]. Annually 2.6 million stillbirths occur worldwide with 98% of them occurring in developing countries [2, 3]. Intrapartum-related events are now the third leading cause of all deaths among children under age 5 years with nearly half of newborn deaths taking place during the first day of life [4]. The low proportion of intrapartum stillbirths in high-income countries suggests that intrapartum stillbirths are largely preventable with quality intrapartum care including prompt recognition and management of intrapartum complications [5].

Most maternal and newborn deaths are in principle preventable [6]. Progress has been made in the reduction of newborn mortality rates by 47% worldwide between 1990 and 2015, from 36 to 19 deaths per 1000 live births. However, the rate has declined more slowly in most low-resource settings, and is still far from the Sustainable Development Goal target of less than 12 newborn deaths per 1000 live births by 2030 [7, 8]. In 2014, the Every Newborn Action Plan, a global multi-partner movement to end preventable maternal and newborn deaths and stillbirths, set a target for national stillbirth rates of 12 or fewer stillbirths per 1000 births in all countries by 2030, accompanied by action in countries to address disparities [6]. Access to and utilization of high quality, evidence-based intrapartum care is one way to further reduce intrapartum death rates [5, 9, 10].

It is widely accepted that the intrapartum stillbirth rates of a hospital or country is reflective of the quality of care received during labor, and newborn mortality in the 24 h following birth can reflect the quality of care during labor as well as immediate postnatal care. Quality of maternity care is often limited by gaps in individual clinicians, nurses/midwives’ knowledge and skills, as well as systems level issues, such as supply and human resource management – staff shortage and maldistribution of health personnel [11–13]. A number of surveys in developing countries have shown that the majority of healthcare facilities in low and middle income settings, although designated to provide either basic or comprehensive emergency obstetric and newborn care (EmONC), may be unable to do so [14–16]. In many cases, structures are in place, and equipment and consumables are reported to be available, but the staffs reported that they lacked competency and skills and were therefore unable to provide all the signal functions of EmONC and essential newborn care [14, 16, 17]. In other instances, the lack of knowledge and skills to provide EmONC is compounded by non-utilization of simple but proven health technologies and equipment [18, 19].

Skills competency-based training in skilled birth attendance, emergency obstetric and early newborn care is an approach that is successful in improving knowledge and skills [20]. Skilled health personnel trained to International Confederation of Midwives (ICM) standards can provide nearly all of the essential care needed for women and newborns. This includes performing all signal functions of emergency maternal and newborn care to optimize the health and well-being of women and newborns [21]. Insufficient pre-service and in-service training is a key barrier to provision of quality EmONC services [22–24]. This therefore calls for the need to build the capacity of healthcare providers to recognize and manage complications during pregnancy, childbirth and the post-partum period. In-service training for skilled birth attendants is one of the most common interventions to address lack of knowledge and skills and improve health outcomes [20, 25]. Targeted, repeated training and/or mentorship approaches at the job site are preferable to one-time training and lead to the use of new skills in the clinical setting. Evidence suggests that these approaches are associated with greater improvement in clinical performance, knowledge and skills retention and health outcomes [11, 20, 26–29]. However, there is little evidence generated on the utilization and applicability of these mentorship and on-the-job training methods, which can be instrumental in maintaining the skills and competence in areas trained in Kenya [13].

Kenya’s perinatal mortality, defined as the pregnancy losses occurring after seven completed months of gestation (stillbirths) plus deaths to live births within the first 7 days of life (early neonatal deaths) is 29 per 1000 live births [30]. Two major causes of newborn deaths in Kenya, birth asphyxia and sepsis could be prevented by increasing the use of evidence-based interventions during labor, birth and the immediate postnatal period [31]. The Kenya Health Policy 2012–2030 and the Kenya health sector strategic and investment plan 2012–2017 describe the country’s goal to reduce institutional newborn mortality from 31 to 13 per 1000 live births by 2030 through training of skilled birth attendants in basic emergency obstetrics and newborn care and essential newborn care [32, 33].

Busia County Government’s Department of Health implemented a low-dose high-frequency (LDHF) facility-level focused mentorship program with support from the United Kingdom’s Department for International Development (DfID) Boresha project implemented by Save the Children International in the main referral facilities for staffs in maternity and mother & child health (MCH) departments targeting to reduce maternal and neonatal mortalities. Using this approach for in-service training, healthcare workers were mentored on key evidence-based intrapartum and immediate newborn care practices, using current national/global guidelines on job site. The indicators of change in practice and health outcomes after mentorship in a busy basic emergency obstetrics and newborn care (BEmONC) facility providing midwife-led maternity services were evaluated using the adapted Kirkpatrick framework for evaluating a training program. This framework has four distinct levels of training evaluation: level 1 (reaction – acceptance to mentorship), level 2 (learning – change in knowledge and skills), level 3 (behavior – change in behavior and practice) and level 4 (results – health outcomes and change in perinatal mortalities) [34, 35]. Evaluation of the effectiveness of LDHF mentorship targeted the Kirkpatrick’s levels 3 and 4 focusing on the change in practice and health outcomes. Multiple training/mentorship interventions implemented by several partners in the same group of healthcare providers have focused on evaluating levels 1 and 2 [20]. This study hypothesized that the LDHF mentorship approach to improving clinical skills would lead to an increase in assisted vaginal deliveries and overall reduction in institutional intrapartum stillbirths and early neonatal mortality in a busy rural BEmONC facility in Busia County.

## Methods

### Design and setting

This was a nonequivalent control group pretest – posttest design, a quasi-experimental design that involves an experimental treatment and two groups (study and control) of subjects, not randomly assigned, observed before and after its implementation [36]. The experimental treatment was the low-dose high-frequency mentorship while the two groups were a study hospital where mentorship implemented by the project was provided and a control hospital where no intervention was provided. Both hospitals were reviewed before as well as during the mentorship, to test if the LDHF mentorship made any difference in the practice and perinatal outcomes. Data was reviewed at two similar period points. The pre-intervention period reviewed was Oct 2015 to July 2016 while the post-intervention period was August 2016 to May 2017 (last 10 months of the intervention before the protracted nurses strike that paralyzed operations in all government facilities for 6 months from June 2017) in a high-volume referral hospital. The mentorship program started in August 2016 and ended in May 2017 in the county and at the study hospital. The hospitals were optimally functioning during the periods reviewed in this study. The mentorship focused on the mode of delivery, newborn resuscitation and referral for delivery services as the indicators of change in practice and change in health outcomes after training/mentorship as study designs involving direct observation of practice may be expensive and underpowered [20]. These are routine indicators captured and reported by the healthcare workers in the national MOH registers.

The study hospital was Nambale subcounty hospital from Nambale subcounty while the control hospital was Khunyangu subcounty hospital from Butula subcounty, two subcounties out of the seven that make up Busia county in Western Kenya region. This region had a high perinatal mortality rate (26 per 1000 live births vs the country’s average of 29 as per the 2014 KDHS report). The control hospital was from one of the three subcounties that were not part of the maternal and newborn health project implementation by Save the Children in Busia county. The two hospitals had similar characteristics (level, staffing, infrastructure and health services) as they were all level 3 health facilities and provided midwifery-led maternity services [32, 33]. The study and control hospitals are the largest BEmONC referral facilities in their respective subcounties and operate as level 3 facilities providing basic emergency obstetrics and newborn care. The study and control hospitals recorded an average of 1100 and 1400 deliveries respectively per annum as at 2017. These were ideal sites to assess the difference or effectiveness of a LDHF mentorship approach as they provided 24-h coverage per day and recorded the highest deliveries compared to other facilities in the same level in the county. Secondly, the maternity services at these facilities are midwife-led and the facilities offer other integrated comprehensive reproductive health services including cervical cancer screening, prevention of mother to child transmission of HIV/AIDs (PMTCT) and laboratory services. In addition, these facilities are training/learning centres for trainee nurses as well as clinical offers in the region. Importantly, the hospitals offer both promotive and preventive care as well as curative care with majority of the population selecting them as their primary health facilities.

### Procedures

#### Participants

Clinical mentors were highly experienced skilled birth attendants who were voluntarily working as champions and managers of maternal and newborn health care in their respective subcounties/facilities. A team of three clinical mentors facilitated the mentorship sessions in the facility (and the subcounty). A clinical mentor (also a master trainer) was either a doctor, nurse/midwife or reproductive health clinical officer from high volume facilities which were defined as a facility with an average of five or more deliveries in a day. They were all trainers of trainees (ToTs) in Emergency Obstetric and Neonatal Care (EmONC), Essential Newborn Care (ENC) and Kangaroo Mother Care (KMC). Additionally, they had been formally trained in clinical mentorship including the components of mentoring, Benner’s stages of clinical competence (from novice to expert), stages of mentoring process and the models of mentorship. They were integrated into the MOH’s subcounty supervisory team to provide ongoing, on-site individual mentorship to dispensary, health centre, subcounty hospital and county referral hospital healthcare providers and to drive systems-level quality improvement activities. A total of 13 nurses/midwives providing antenatal, intrapartum and immediate postpartum care received mentoring at the facility. The seven BEmONC signal functions mentored on were as in Table 1. Other key areas covered were: antepartum care including danger signs and pregnancy-related complications, management of second stage of labor – correct and consistent use of the partograph in monitoring labor, management of prolonged & obstructed labor, conducting complicated vaginal deliveries – breech and assisted vaginal deliveries (by vacuum extraction); active management of the third stage of labor, use of uterine balloon tamponade in management of postpartum hemorrhage, immediate care of the newborn, kangaroo mother care and infection prevention during labor and child birth. The project facilitated a lunch and transport allowance of two thousand Kenyan shillings per day for the planned sessions per mentor. Individualized unstructured on-job training sessions to strengthen key skills learned were conducted voluntarily by the mentors without compensation. Mentees were not financially compensated in any way to participate as this would provide challenges with the sustainability of the process despite its benefits. However, mentees identified for their new expertise were recognized as champions within the health facility to promote on-job training for fellow colleagues and trainee nurses.

**Table 1:** Basic emergency obstetric and newborn care package (signal functions)

	Signal functions
1	Administration of IV antibiotics
2	Administration of magnesium sulphate
3	Administration of parenteral oxytocics
4	Performing manual removal of the placenta
5	Performing removal of retained products
6	Performing assisted vaginal delivery (e.g. by vacuum extraction)
7	Performing newborn resuscitation

#### Equipment, protocols and guidelines

Maternity equipment including fetal doppler ultrasound scans, vacuum extractors, neonatal resuscitation equipment (bag and valve masks, penguin suction device) and infection prevention and control equipment were replenished. This was to supplement the available technology and improve functionality at the facility. In addition, MOH guidelines – national guidelines for quality obstetrics and perinatal care (2012), basic pediatric protocols for ages up to 5 years – 4th edition (2016) etc. to support knowledge and skills were disseminated to the facility. MamaNatalie® and NeoNatalie® simulators were given to the facility to enable regular practice of targeted skills.

#### Mentorship program

Clinical mentorship was a sustained, collaborative relationship in which a highly experienced health care provider guided improvement in the quality of care delivered by other providers and the health care systems in which they work. Two models of mentorship were implemented: Primary healthcare based mentorship and telephone mentorship [37]. The low-dose sessions included competency acquisition through simulation, case-based learning, and small content packages spread over short time intervals. In primary health care based clinical mentorship (on site mentorship) model, mentees were assigned to mentors who visited them in their designated work stations. During the visits, mentors observed the mentees as they attend to the patient or handle complications and then provide an opportunity for reflection and feedback after observation. This was followed by structured continuous professional education on the related areas. Importantly, both individual (individual mentee vs mentor) and group mentorship (several mentees: 2–6 vs at least 2 mentors) sessions were conducted. Individual mentorship was applicable to an individual mentee’s specific gap in knowledge and/or skill in a particular EmONC area. Group mentorship was commonly applicable where the knowledge and skill in question was meant for a blend of healthcare providers at a specific point. Secondly, telephone conversations and text messaging where both the mentor and the mentees used telephone conversations and text messaging for clinical questions, case reviews and referrals. This model was used by mentees when consulting the mentors for support on how to handle complicated cases. Clinical mentors also used this model for making follow-ups on the progress of the mentee especially after the initial intense stage.

#### Timeline and frequency for mentorship

The amount of time of engagement between the mentor and mentee to transfer knowledge and skills varied greatly depending on the mastery and retention of skills. The duration of mentorship for each mentee was at least a month and no more than 6 months. An average of four mentor vs mentee contacts per knowledge/skills demonstrations were conducted. However, this was evaluated on case by case basis depending on the acquired knowledge and mastery of the skill. Intensive support was given in the first month with fewer contacts in the subsequent months. The decision to reduce the intensity was made by the team on the general assessment of the progress of the mentee after each phase. During the first month, the mentor visited the mentee at the work place once a week for 3 weeks in addition to telephone communication and text reminders. In the second month, the mentor visited the mentee once fortnightly and then the 3rd to 6th month was done monthly. The mentors ensured that the mentees examined, treated and managed the obstetric and neonatal emergencies. This was done with dignity and respect after seeking informed consent and as per the hospital’s protocols of care. As the mentee gained confidence and competence in managing obstetric and neonatal emergencies, he/she required less face to face interaction with the clinical mentor and in such cases, phone consultation was used as a primary means of support. The clinical mentors determined the mentee’s competency on essential obstetric and neonatal emergencies knowledge and skills using standard practice checklists, observed deliveries and interviews on case management processes applying Benner’s 5 – chronological stages of clinical competence (novice, beginner, competent, proficient and expert) [38]. If the mentee required further support, contact sessions were planned and continued as required (Table 2).

**Table 2:** Components of low-dose, high-frequency mentorship approach

	Components
1	Supply of newborn resuscitation equipment, fetal doppler ultrasound scans, vacuum extractors, delivery sets and obstetric and perinatal care national guidelines at first low-dose training session
2	Identification of individual skilled birth attendant skills gap in antepartum, intrapartum & postpartum care
3	Two 2-day low-dose simulation sessions (for facility skilled birth attendants) in the 1st and part of 2nd week as applicable
4	High-frequency practice sessions using partographs, MamaNatalie® and NeoNatalie® anatomic models, bag and mask for resuscitation, vacuum extractors etc. from the 2nd week
5	Mentoring calls/conversations between mentors and mentee including reminder messages
6	Systematic and structured supportive supervision for mentees by the mentorship team
7	Competence evaluation applying Benner’s stages of clinical competence

#### Data collection

Data was collected using the MOH tools available at the health facility level – MOH 333 (Maternity register that provides a daily activity of deliveries and their outcomes including obstetric and perinatal complications) and MOH 711 summary report (Integrated Summary Report: Reproductive & Child health, Medical and Rehabilitative Services). To improve data quality, data strengthening efforts including documentation and verification was routinely done by the unit’s nurses/midwives and facility’s health records and information officer daily to ensure that all cases were captured in the relevant MOH registers. Data from the health facilities were summarized and routinely entered in the national District Health Information System (DHIS2) by the Subcounty’s Health Records Information Officer.

#### Data analysis

STATA version 12 was used for the analysis. The findings for the indicators of interest from the two time periods were compared through a two-sample test of proportions, and MOH summary data and program reports were also reviewed to triangulate the information obtained from the DHIS2. Proportions and *p*-values were reported to test the strength of the evidence after the intervention.

## Results

### Mode of delivery

Spontaneous vaginal delivery was the commonest route of delivery between 2015 and 2017 in the two facilities. Assisted vaginal deliveries (vacuum extractions) increased 13 times from 0.2 to 2.5% during the implementation period (*P* < 0.0001) at the intervention facility with no change at the control facility. Breech deliveries halved from 1.5 to 0.7% (*P* = 0.0475) in the mentorship facility with no significant change at the control facility. Importantly, vacuum extractions were conducted due to delayed/prolonged second stage and/or non-reassuring fetal-maternal domains in second stage with adequate cephalopelvic proportions to improve fetal outcomes. Overall, there was a reduction in the proportion of normal deliveries in the two facilities and an increase in assisted vaginal deliveries (vacuum extractions) at the intervention facility and an increase in breech deliveries at the control facility between the two periods.

There was a double increase in the proportion of obstetric cases referred to a comprehensive emergency obstetric and newborn care facility for caesarean delivery from 3.0% (34 out of 1118) to 6.5%, (76 out of 1172), *P* < 0.0001, from the intervention facility. These deliveries occurred at a different birthing center, and so the outcomes of these infants were not part of the analysis. In the control facility, there was no observable change 3.1% (40 out of 1291) vs 3.1% (36 out of 1151), *P* = 0.4834, in the proportion of cases referred for caesarean delivery during the same period. (See Table 3).

**Table 3:** Mode of delivery between pre-intervention (Oct 2015-July 2016) and during/after intervention (Aug 2016-May 2017)

	Intervention Hospital			Control Hospital		
Mode of Delivery	Pre [n (%)] (*n* = 1084)	Post [n (%)] (*n* = 1096)	*P*-value	Pre [n (%)] (*n* = 1275)	Post [n (%)] (*n* = 1140)	*P*-value
Normal deliveries	1066 (98.3)	1061 (96.8)	0.0100*	1251 (98.1)	1115 (97.8)	0.2944
Breech deliveries	16 (1.5)	8 (0.7)	0.0475*	23 (1.8)	24 (2.1)	0.2963
Assisted vaginal deliveries (vacuum extraction)	2 (0.2)	27 (2.5)	<0.0001*	1 (0.1)	1 (0.1)	0.4684

*P ≤ 0.05 statistically significant

### Perinatal mortality outcomes

There was a marginal increase in the proportion of live births between the two periods (98.9% vs 99.3%, *P* = 0.1758) at the intervention hospital with a clinically significant reduction in the same proportions at the control hospital (98.0% vs 97.0%, *P* = 0.0547). Although the proportion of babies born with low APGAR scores requiring newborn resuscitation doubled from 1.7 to 3.7% (*P* = 0.0021), the proportion of neonatal deaths almost halved from 0.5 to 0.3% (*P* = 0.2313) over the same period at the intervention hospital. Over the course of the intervention, the proportion of fresh stillbirths significantly decreased 5 times from 0.5 to 0.1% (*P* = 0.0491) with no change in the proportion of macerated stillbirths (0.6% vs 0.6%, *P* = 0.4891) at the intervention hospital. However, there was a triple increase in the proportion of macerated stillbirths (0.4% vs 1.4%, *P* = 0.0039) with no change in the proportion of babies born with low APGAR scores, fresh stillbirths and neonatal deaths at the control hospital (*P* > 0.05) (See Table 4). *There were no maternal mortalities reported in the two facilities during the two study periods*.

**Table 4:** Perinatal outcomes between pre-intervention (Oct 2015 – July 2016) and during/after intervention (Aug 2016 – May 2017)

	Intervention Hospital			Control Hospital		
Perinatal Outcomes	Pre [n (%)] (*n* = 1086)	Post [n (%)] (*n* = 1102)	*P* – value	Pre [n (%)] (*n* = 1272)	Post [n (%)] (*n* = 1144)	*P* – value
Live births	1074 (98.9)	1094 (99.3)	0.1758	1247 (98.0)	1110 (97.0)	0.0547*
Babies born with low APGAR scores ^a^	18 (1.7)	40 (3.7)	0.0021*	4 (0.3)	4 (0.4)	0.4345
Macerated stillbirths	7 (0.6)	7 (0.6)	0.4891	5 (0.4)	16 (1.4)	0.0039*
Fresh stillbirths	5 (0.5)	1 (0.1)	0.0491*	20 (1.6)	18 (1.6)	0.4991
Neonatal deaths ^a^	5 (0.5)	3 (0.3)	0.2313	4 (0.3)	1 (0.1)	0.1122

^a^The denominators for this analysis are 1074 and 1094 for the mentorship facility and 1247 and 1110 for the control facility for Oct’15 – July’16 and Aug’16 – May’17 periods respectively

*P ≤ 0.05 statistically significant

## Discussion

Mentorship aimed to transform classroom learning to clinical skills, build the capacity of health care workers to promptly respond to obstetric emergencies and promptly identify complications and refer appropriately. This study demonstrated that through this onsite approach, nurse/midwives improved their performance of labor monitoring and identification of mothers in labor at high risk, assisted vaginal (vacuum extractions) deliveries and identification and management of newborns at risk of birth asphyxia. Ultimately, this contributed to a reduction in fresh stillbirths in a midwifery-led health facility. Within an enabling environment, nurse/midwives trained to ICM standards can provide nearly all of the essential care needed including all signal functions of emergency maternal and newborn care to optimize the health and well-being of women and newborns [21].

The busy rural health facilities are acutely understaffed compared to the required minimum as proposed in the Kenya Human Resource for Health norms of at least 20 registered midwives at this level of facilities [39]. This is the case in most low and middle income countries as there is a critical shortage of healthcare providers in Africa; the World Health Organization reported that 36 of the 57 countries facing chronic human resource shortages in the health sector are in sub-Saharan Africa [17] and that only 12.0 per 10,000 people of nursing/midwifery personnel are in Africa compared to 80.5 nurses/midwives per 10,000 people in the European region. Evidence shows that frequent engagement of facility-based health workers in offsite trainings sometimes causes artificial staff shortages and adversely affects service delivery especially in rural areas [40, 41]. This therefore underscores the benefits of implementing the onsite mentorship approach in building the capacity of healthcare workers.

Emergency obstetric and neonatal care (EmONC) is a proxy indicator for monitoring maternal and perinatal mortalities, even though data on this care is rarely available [42]. Results of an assessment of 13 Kenyan counties with the highest maternal mortality in the health facility readiness to provide EmONC report revealed that health workers deployed in the maternity and newborn departments who had received training in EmONC was low, ranging between 10% and 30% in most counties [16]. This onsite mentorship dubbed the low-dose, high-frequency training approach has the potential to build the capacity of a critical mass of healthcare providers working in these units in the key maternal and newborn life-saving skills. This is emphasized by the Congo study that recommended that to reduce maternal and perinatal mortality, it is essential that staff skills regarding EmONC be strengthened [42].

Normal spontaneous vaginal delivery was the commonest route of delivery at both the intervention and control hospital. This could be due to the fact that this level of facilities handle low-risk pregnancy and labor as recommended by WHO [43]. However, it is estimated that 15% of pregnant women will develop a complication during pregnancy, childbirth or the puerperium, which will require EmONC and therefore the need to have a ready environment and skilled birth attendants to prevent or manage an obstetric emergency at all times [44].

There was a significant increase in the proportion of assisted vaginal deliveries (vacuum extractions) at the intervention hospital (with no change at the control hospital) during the mentorship period compared to the previous period despite availability of equipment. This could be attributed to the mentoring the healthcare workers received to improve their capacity to conduct vacuum extractions. This clearly strengthens the evidence that health facility readiness for providing EmONC services requires staff with the capacity to perform the obstetric life-saving skill for the mother and baby. This finding is consistent with the poor service availability and readiness in provision of vacuum extractions in surveys conducted in the country and other African countries [14, 16]. In addition, evidence shows that this rate is less than the 5% uptake of vacuum deliveries in low resource settings [45]. When spontaneous vaginal delivery in the second stage of labor is not a possibility, operative vaginal delivery may be a safe, acceptable alternative to cesarean delivery [46]. Both the American College and the Royal College of Obstetricians and Gynecologists continue to support the use of vacuum and strongly encourage residency programs to incorporate the teaching of these skills into their curricula [47]. In resource-limited settings where few CEmONC facilities exist serving more than the recommended WHO population of 500, 000 per facility [43], training the nurses/midwives in vacuum extractions could provide the alternative best option of care for mothers with delayed second stage of labor.

Breech deliveries reduced significantly at the intervention hospital with no change in the control hospital. Breech presentations in the first stage of labor and/or in primigravida women are considered high risk pregnancies/presentations to be referred to a facility that offers comprehensive emergency obstetrics and neonatal care in the country for cesarean section [31]. Non-cephalic presentations are the greatest fetal risk factors associated with stillbirths [48, 49]. Secondly, breech deliveries are dependent on the appropriate indication and therefore lack of patients with appropriate indications are reasons for non-performance of some EMONC signal functions as documented [24]. These being BEmONC facilities, breech deliveries can only be conducted if breech presentation cases are presented at the facilities in second stage of labor. Mentorship ensured that skilled birth attendants had the requisite ‘know-do’ capacity to perform the abnormal vaginal delivery making the complete environment for provision of EmONC services.

The proportion of babies born with low APGAR scores doubled at the intervention facility with no change in the control facility. Conversely, the neonatal deaths clinically reduced at both the intervention and control facility. This could potentially be due to appropriate identification by healthcare workers that many depressed newborns can be revived through provision of resuscitative measures which were less robust prior to the mentoring [50]. However, other factors outside the perinatal care are contributors to neonatal mortality in the region [51]. When assigned correctly, the APGAR score is the best quick method to discriminate between infants who require resuscitation at birth and those who do not; to predict outcome; and to evaluate change in the condition of the newly born over the first minutes of life [52, 53]. Theoretically, the onsite mentorship aimed to transform classroom learning to clinical skills, build the capacity of healthcare workers to promptly respond to obstetric emergencies and promptly identify complications and refer appropriately reducing the institutional delay 3 [54]. This approach improved the capacity of the healthcare workers to identify the newborns at risk during the first and the second stages of labor and prepare accordingly for the simple life-saving resuscitative interventions.

There were significant reductions in the proportion of fresh stillbirths (and neonatal deaths) at the intervention facility with no change at the control facility. This could be explained by the lessened delays experienced during referrals in search of cesarean sections at the higher CEmONC facility. Besides, the improvement observed in the referral of high-risk pregnancies/labor to a comprehensive facility is a key contributor to the reduction of stillbirths. This could be partly be due to the fact that this level of facility handles low-risk pregnancy and labor as recommended by WHO [43]. Besides, improved labor monitoring through partograph and uptake of operative vacuum deliveries in prolonged second stage of labor minimizes the delays in second stage that are predictors of poor neonatal outcomes [49, 55]. It has been suggested that the neonatal morbidity once associated with operative vaginal delivery may actually be a function of an abnormal labor process itself, rather than a consequence of an operative vaginal intervention [47]. This provides the first line of emergency care as shown by the 2013 service availability and readiness assessment mapping (SARAM) of emergency obstetric care in Kenya. Caesarean section for obstetric emergencies is available in only 1% of these first entry point into the health tier system for most pregnant mothers in Kenya [56]. In addition, with at least good monitoring of the cervical dilation and descent during labor observed, most fresh stillbirths associated with poor labor monitoring are averted [57]. Our findings are similar to a 2013 study by Msemo et al. in nine Tanzania hospitals which showed that provision of newborn resuscitation - specifically, by skilled birth attendants trained in Helping Babies Breathe (HBB) - reduced neonatal mortality by 47% [58]. Evidence from a systematic meta-analysis by Lee and colleagues also demonstrated that effective newborn resuscitation at birth can prevent asphyxia – related newborn deaths by 30% [59]. Building the capacity of nurses/midwives in these simple interventions to improve the quality of essential newborn care – importantly, improving steps to help newborns breathe at birth are life-saving [1, 10]. Consequently, these interventions offer the best option of reducing perinatal deaths in the local settings where nurses/midwives provide services to the majority of Kenyans who access MNH services via public sector primary health facilities (dispensaries and health centres) that constitute 75% of public sector facilities in Kenya [13].

There was a double increase in the proportion of cases referred for comprehensive emergency obstetrics and newborn care from the intervention hospital with no change at the control hospital. This approach of training ensured that as part of the skilled health professional, midwives acquired competencies to facilitate physiological processes during labour and delivery to ensure a clean and positive childbirth experience and identify and manage or refer women (and/or newborns) with complications in pregnancy and labor [21]. The increase in the proportion of macerated stillbirths at the control facility with no change at the intervention facility could partly be part of the referral as part of skilled management. The mentorship program ensured improved knowledge on danger signs in pregnancy and labor for mentees. This therefore translates into improved client care and health education to improve pregnancy outcomes for women.

The regular and structured clinical mentorship model using subcounty trainers and county support supervisors was found to be an efficient and expedient way to train nurses/midwives in their local setting. However, it must be understood that effective mentorship begins with the organizational culture and must have organizational buy-in to be successful [28]. This model allowed the mentees to consult, review, share challenges and successes with the mentorship teams on regular basis. This systematic skill-building, regular and consistent supportive supervision is critical to the success of access to and provision of timely and high quality emergency obstetrics and newborn care in poorly-resourced maternity setups [23]. Evidence reported in a multi-country study showed that regular short-interval (less than 6 months) structured monitoring and support to healthcare workers in emergency life-saving skills through supportive supervision promotes knowledge and skills retention in clinical settings [60]. When founded on supported longer-term mentor-mentee relationships, mentorship enables the development of competent practice [11]. This onsite mentorship approach can complement the typical pre-service nurse/midwife training that is largely didactic despite being the main providers of maternity care [20, 61]. One study in Kenya showed that the average duration of neonatal resuscitation training was 3 h with 50% of the healthcare providers (HCPs) having missed out on practical exposure [22]. The passive classroom didactic instruction often results in improved knowledge but no improvement in clinical practice as compared to the use of interactive techniques such as clinical simulation, case-based learning, hands-on practice with anatomic models, and immediate feedback on performance [62].

This study has its strengths and limitations. The clinical mentorship intervention was implemented in six health facilities in Busia County but this study focuses on one facility – Nambale subcounty hospital. The selection of Nambale subcounty hospital to implement the clinical mentorship activities was largely focused on a facility providing basic EmONC services with greatest need and serving remote areas of the Busia County in the spirit of the project’s objectives. Preliminary findings from the other five CEmONC hospitals (results not included in this paper) showed no differences in fresh stillbirth rates as they mainly handle high-risk and/or referral cases that are dependent on other factors not assessed by this paper. Use of local mentors in the facility/subcounty for onsite mentorship underscores the importance of promoting sustainability of the intervention. Use of a control hospital with similar characteristics underscores the contribution and credibility of the mentorship approach findings to change in pregnancy and childbirth practice and maternal and neonatal health outcomes. The small samples and short time period of intervention means that these findings should be interpreted within the local context. In addition, this study did not take into consideration the individual cause of the stillbirths and newborn deaths as it focused on the overall outcomes since the goal of mentorship was to foster improved institutional standardized practice on mother and baby life-saving interventions. Use of DHIS2 data is likely to have quality issues (timeliness, accuracy and completeness). However, the project team supported the facility and subcounty teams conduct verifications with individual health facilities before entering in the health information system.

## Conclusions

The LDHF mentorship approach improved the competencies of midwives to identify, manage and/or refer pregnancy and childbirth cases and/or complications including the signal functions of emergency maternal and newborn care to optimize the health and well-being of women and newborns. Midwife-led maternity services have the capacity to reduce institutional intrapartum perinatal deaths and foster sustained retention of critical EmONC skills when closely supported through targeted quality low-dose high frequency life-saving onsite mentorships and supportive supervision. Building the capacity of midwives through sustainable onsite mechanisms provides the first line of defense in handling obstetric emergencies through improved decision-making and provision of quality obstetrics care in low-resourced settings improving emergency services availability and readiness.

### Recommendations

Our findings call for the following key recommendations: (1) There is need to scale this training approach to lower level facilities that experience chronic staff shortages and know-do gaps in midwife-led maternity services to improve the life-saving skills for mothers and newborns; (2) Counties should invest more resources in strengthening and sustaining the LDHF trainings/mentorships so that health facilities are readily prepared to provide the emergency life-saving signal functions.

## Data Availability

The datasets used and/or analysed during the current study are available from the corresponding author on reasonable request. The data was extracted from the Kenya Health Information System (KHIS), formerly the District Health Information System 2 (DHIS2), an open source public access system where all MOH reporting is done and requires registration credentials to access. The link to the databases used is https://hiskenya.org/dhis-web-pivot/.

## Abbreviations

BEmONC: Basic Emergency Obstetrics and Newborn Care
CEmONC: Comprehensive Emergency Obstetrics and Newborn Care
DHIS: District Health Information System
EmONC: Emergency Obstetrics and Newborn Care
ENC: Essential Newborn Care
ICM: International Confederation of Midwives
KMC: Kangaroo Mother Care
LDHF: Low-dose high-frequency
MOH: Ministry of Health
WHO: World Health Organization
